# Increased intraepithelial CD3+ T-lymphocytes and high PD-L1 expression on tumor cells are associated with a favorable prognosis in esophageal squamous cell carcinoma and allow prognostic immunogenic subgrouping

**DOI:** 10.18632/oncotarget.18606

**Published:** 2017-06-22

**Authors:** Moritz Jesinghaus, Katja Steiger, Julia Slotta-Huspenina, Enken Drecoll, Nicole Pfarr, Petra Meyer, Björn Konukiewitz, Marcus Bettstetter, Kathrin Wieczorek, Katja Ott, Markus Feith, Rupert Langer, Wilko Weichert, Katja Specht, Melanie Boxberg

**Affiliations:** ^1^ Institute of Pathology, Technical University of Munich, Munich, Germany; ^2^ Molecular Pathology South-Bavaria, Munich, Germany; ^3^ Institute of Pathology, University of Heidelberg, Heidelberg, Germany; ^4^ RoMED Surgical Clinic, Rosenheim, Germany; ^5^ Department of Surgery, Technical University of Munich, Munich, Germany; ^6^ Institute of Pathology, University of Bern, Bern, Switzerland; ^7^ German Cancer Consortium, Partner Site Munich, Munich, Germany

**Keywords:** esophageal squamous cell carcinoma, immunologic microenvironment, PD-L1, tumor infiltrating lymphocytes, intraepithelial CD3, Pathology Section

## Abstract

Esophageal squamous cell carcinoma (ESCC) is the most common esophageal cancer associated with poor prognosis and additional therapeutic strategies must be implemented to optimize ESCC treatment. Meanwhile, the important biologic role and potential prognostic and therapeutic implications of a tumors immunologic microenvironment (IM) have been recognized in various cancers.

In order to investigate the contexture and the prognostic relevance of the IM in ESCC, we immunohistochemically evaluated the extent of overall/intraepithelial TILs (CD3+/CD8+) and of PD-1 / PD-L1 expression in a cohort of 125 therapy-naive ESCCs, additionally assessing *PD-L1* copy number status via fluorescence in-situ hybridization.

High intraepithelial CD3+ TILs (CD3ihigh) and high PD-L1 expression on tumor cells (PD-L1high) were each significantly associated with improved overall- (OS) (CD3+: *p* = 0.019; PD-L1: *p* = 0.028), disease specific- (DSS) (CD3+: *p* = 0.05; PD-L1: *p* = 0.006) and disease free survival (DFS) (CD3+: *p* = 0.009; PD-L1: *p* < 0.001). CD3ihigh- and PD-L1high cases were significantly associated with one another (*p* < 0.001). Subgrouping of ESCC revealed decreased OS (*p* = 0.031), DSS (*p* = 0.012) and DFS (*p* < 0.001) for CD3ilow/PD-L1low cancers.

Our data not only associate CD3ihigh- and PD-L1high ESCC with a beneficial outcome, but also demonstrate PD-L1high- and CD3ihigh status to be closely intertwined. Furthermore, our study demarcates a prognostically unfavorable, “non-immunoreactive” CD3ilow / PD-L1low ESCC-subgroup, potentially forming the basis for an immune-based stratification of ESCC.

## INTRODUCTION

Esophageal squamous cell carcinoma (ESCC) is a substantial cause of cancer-related death worldwide, accounting for approximately 80% of esophageal cancers [1–6]. ESCC is usually associated with poor patient prognosis, with reported 5-year overall survival rates varying from 15-40% [2, 4, 7]. Although diagnostic and therapeutic advances have led to slight improvements in the clinical management and outcome of ESCC [2, 5, 7], the implementation of additional treatment strategies and novel prognostic biomarkers must be expedited to optimize ESCC treatment.

The recognition of the immunologic microenvironment (IM) of a malignant tumor as a potentially powerful biomarker of prognostic and therapeutic relevance has led to a resurgence of immune based therapy strategies in solid cancers. Firstly, the immunologic tumor-host relationship appears to be of substantial importance for the carcinogenic process [8, 9] and especially the extent of CD3+ and CD8+ tumor infiltrating lymphocytes (TILs) seems to be of special interest for the evaluation of a potential immunogenic antitumor response [10–13]. Particularly, intraepithelial CD3+ T-cells have been identified as valid prognosticators in a variety of adenocarcinomas [14–17]. Secondly, immune-checkpoint blockade, especially via inhibition of the potent immunoevasive effects of the Programmed Death-1 (PD-1) / Programmed Death – Ligand 1 (PD-L1) axis, has become a powerful tool of tumor immunotherapy, with variable responses in diverse cancer types [18, 19, 20] and partially conflicting results regarding the predictive value of immunohistochemical PD-L1 expression [18]. Furthermore, a recent pan-cancer classification approach proposes a model of four different types of tumor microenvironments, based on a tumors T-cell infiltration and PD-L1 positivity [13].

However, in ESCC, data regarding its IM are limited and the prognostic value of PD-L1 expression is still a matter of debate, as some studies associate PD-L1 expression with a rather favorable [21, 22] prognosis, while others postulate a less favorable [23–25] disease course for PD-L1 positive cancers.

In order to investigate the contexture and possible clinicopathological implications of the IM including the PD-1 / PD-L1 axis in ESCC, we immunohistochemically analyzed the extent and distribution of overall and intraepithelial CD3+ / CD8+ TILs as well as PD-1 / PD-L1 expression in a tumor series of 125 primary resected, therapy-naive ESCCs. Additionally, the tumors *PD-L1* copy number status was evaluated through Fluorescence in situ Hybridization (FISH).

The questions we addressed focused on *(1)* the specific composition of the IM in ESCC, *(2)* the identification of possible associations of overall and intraepithelial TILs with specific clinicopathological and survival parameters, *(3)* the predictive value of PD-L1 immunohistochemistry in ESCC and (4) the relationship of the PD-1 / PD-L1 axis to the IM, particularly focusing on potential immunologic ESCC subgroups based on their extent of T-cell infiltration and PD-L1 positivity.

## RESULTS

### Clinicopathological characteristics

95 of the 125 ESCC patients in our tumor series were male (76%), 30 were female (24%). Mean age at diagnosis was 60 years (range: 39-83). Rather locally advanced cancers (pT2-pT4: 70/125; 56%) and early stage carcinomas (pT1: 55/125; 44%) were roughly evenly distributed, synchronous lymph node metastases (pN+) were detectable in 57 cases (46%) and four cases showed synchronous distant metastases (pM1: 3%). According to the WHO classification of the digestive system [26], most ESCCs were moderately (G2; 49%) or poorly differentiated (G3; 46%) with only a minor subset of well differentiated tumors (G1; 5%). 53 (42%) patients suffered from local and/or distant relapse and 79 (63%) patients died during follow up, of which 82% (65/79) were tumor specific deaths. Mean follow-up time for patients alive at the endpoint of overall survival analysis was 65.09 months (Table 1).

**Table 1:** Association of immunological and clinicopathological factors with survival parameters (univariate)

		Overall	Events (OS)	Mean overall survival (SE)	*p*-value	Events (DSS)	Mean disease specific survival (SE)	*p*-value	Events (DFS)	Mean disease free survival (SE)	*p*-value
											
		125	79	76.9 (7.1)		65	89.0 (7.9)		53	86.6 (7.4)	
Age					*0.447*			*0.401*			*0.710*
	median and below	68	45	77.9 (8.9)		36	89.6 (10.0)		33	82.4 (8.7)	
	above median	57	35	57.6 (6.6)		29	65.3 (7.0)		20	77.4 (7.4)	
Sex					*0.140*			*0.067*			*0.192*
	male	95	64	73.1 (8.0)		54	82.3 (8.8)		43	82.4 (8.2)	
	female	30	16	72.4 (8.3)		11	83.7 (8.6)		10	83.8 (8.8)	
pT					*<0.001*			*<0.001*		mean DFS not reached	*0.002*
	1	55	26	106.2 (11.5)		31	119.0 (12.2)		15		
	2	40	36	38.9 (5.5)		29	44.9 (6.5)		25		
	3	28	17	76.8 (13.2)		3	86.8 (14.0)		13		
	4	2	1	36.9 (17.7)		2	36.9 (17.7)		0		
pN					*0.012*			*0.014*		mean DFS not reached	*0.005*
	0	68	38	82.8 (8.6)		42	92.9 (9.2)		24		
	1	47	37	60.7 (9.5)		34	74.0 (11.6)		23		
	2	8	3	63.9 (14.0)		3	63.9 (14.0)		4		
	3	2	2	13.9 (4.8)		2	13.9 (4.8)		2		
pM					*0.001*			*<0.001*			*<0.001*
	0	121	76	79.0 (7.2)		61	91.6 (8.1)		49	89.4 (7.5)	
	1	4	4	6.0 (2.8)		4	14.5 (6.0)		4	8.9 (5.6)	
UICC Stage					*0.008*			*0.002*			*<0.001*
	1	51	21	66.6 (6.8)		19	74.7 (7.2)		23	85.7 (7.2)	
	2	49	24	76.8 (10.1)		24	85.6 (11.0)		25	79.5 (9.7)	
	3	21	37	45.1 (7.9)		34	55.6 (8.8)		36	64.1 (9.6)	
	4	4	4	14.5 (16.0)		4	14.5 (6.0)		4	8.9 (5.6)	
Grade (WHO)					*0.039*			*0.071*			*0.402*
	1	6	1	143.2 (19.0)		1	143.2 (19.0)		2	114.2 (24.7)	
	2	61	39	81.2 (9.9)		31	94.3 (11.2)		28	85.2 (9.6)	
	3	58	40	52.5 (6.0)		33	60.1 (6.6)		53	69.6 (7.5)	
CD3i					*0.019*			*0.050*			*0.009*
	low	81	55	62.1 (7.4)		44	73.9 (8.6)		39	78.1 (8.9)	
	high	44	25	96.4 (11.9)		21	106.1 (12.5)		14	104.9 (11.1)	
Distribution of CD3i					*0.007*			*0.003*			*0.004*
	focal	75	54	58.3 (7.1)		46	65.5 (7.8)		37	73.4 (8.9)	
	diffuse	50	26	100.1 (11.8)		19	118.7 (12.6)		16	106.5 (11.1)	
CD8i					*0.163*			*0.486*			*0.185*
	low	87	58	73.3 (8.4)		45	90.3 (9.6)		39	81.5 (8.7)	
	high	38	22	83.5 (10.7)		20	88.0 (11.0)		14	100.3 (12.2)	
Distribution of CD8i					*0.015*			*0.080*			*0.030*
	focal	85	60	61.6 (8.0)		47	74.6 (10.3)		40	70.8 (6.9)	
	diffuse	40	20	94.1 (11.0)		18	98.7 (11.1)		13	110.1 (11.7)	
PD1i					*0.035*			*0.117*			*0.169*
	low	84	53	60.6 (7.4)		42	72.2 (8.7)		35	*80.8 (9.3)*	
	high	41	27	95.5 (12.5)		21	103.2 (13.0)		18	*88.9 (9.0)*	
PD-L1+ TILs					*0.870*			*0.815*			*0.844*
	low	89	56	77.2 (8.6)		47	87.2 (9.5)		38	82.8 (8.4)	
	high	36	23	69.6 (10.7)		18	84.1 (12.4)		15	96.4 (12.8)	
PD-L1 TCs					*0.028*			*0.006*			*<0.001*
	low/absent	87	54	65.0 (8.8)		47	71.7 (9.7)		42	62.4 (6.8)	
	high	38	26	87.8 (9.1)		18	103.8 (10.6)		11	122.4 (10.6)	
Staining intensity of PD-L1					*0.720*			*0.883*			*0.293*
	weak	30	18	55.9 (8.1)		13	68.2 (9.0)		14	60.9 (9.2)	
	intermediate	31	15	73.9 (11.6)		12	97.9 (14.3)		12	117.6 (13.3)	
	strong	28	20	87.1 (13.3)		17	96.1 (12.8)		8	96.8 (14.2)	
PD-L1 copy number status					*0.781*			*0.533*			*0.951*
	Amplification	3	2	25.4 (15.1)		2	25.4 (15.1)		1	37.2 (17.4)	
	Polysomy	19	6	66.2 (10.3)		3	66.2 (10.3)		4	69.2 (11.4)	
	Disomy	92	61	76.5 (8.1)		49	89.2 (9.0)		39	88.5 (8.3)	
	Deletion	10	11	52.5 (10.0)		11	64.4 (10.0)		9	56.5 (11.4)	
CD3i/PD-L1 subgroups					*0.031*			*0.012*			*0.001*
	low/low	60	44	46.8 (5.3)		38	51.6 (5.8)		33	53.8 (6.3)	
	low/high	21	11	76.9 (7.1)		6	117.4 (16.0)		6	113.9 (16.8)	
	high/high	27	15	86.4 (16.0)		12	100.6 (12.8)		5	130.3 (12.7)	
	high/low	17	10	95.4 (21.4)		9	101.4 (22.0)		9	77.4 (14.7)	

### Composition of intraepithelial tumor infiltrating lymphocytes in ESCC

**Figure 1:**
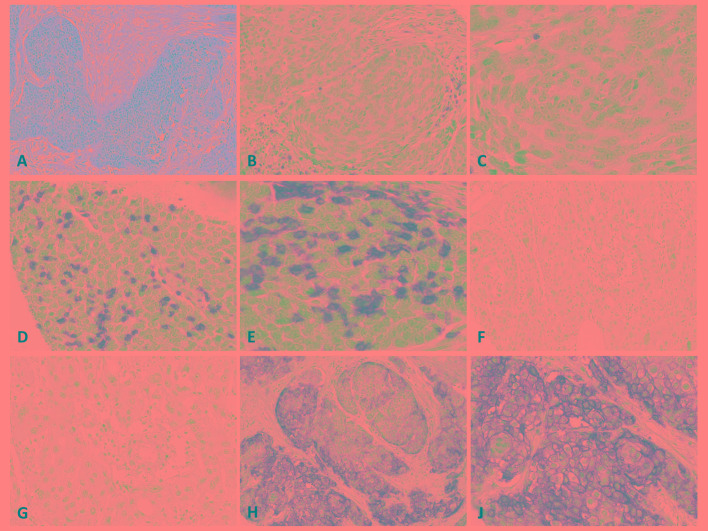
**A**. Hematoxylin-Eosin stain of moderately differentiated ESCC; medium and high magnification of an ESCC with low **B**., **C**. and high **D**., **E**. CD3i count; medium and high magnification of an ESCC without **F**., **G**. and with high PD-L1 expression **H**., **I**..

Intraepithelial CD3+ TIL (CD3i) count was heterogeneous showing a wide range from 0-70/100 (median: 12) tumor cells (TC). 44/125 ESCCs exceeded the 66. percentile, harboring >20 CD3is (43%; CD3ihigh), while the remaining cancers had <20 CD3is and were therefore classified as CD3ilow. 50 tumors (40%) showed diffuse CD3i distribution. Intraepithelial CD8+ TIL (CD8i) count fluctuated from 0-70/100 TCs (median: 6). 38 tumors clustered within the upper third and had >13 CD8is (30%; CD8ihigh). Diffuse CD8is were detectable in 40 (32%) cases. Intraepithelial PD-1+ TIL (PD1i) count varied from 0-25/100 TCs (median: 3) and 41 cases had a high PD1i count of >6 PD1is, exceeding the 66. percentile (33%; PD1ihigh) (Table 1, Figure 1).

### Density of tumor infiltrating lymphocytes in the whole tumor area

Overall density of CD3+ TILs occupying the whole tumor area fluctuated from 1-60% (median: 10%; high overall CD3+ TILs: 15%, 83/125). Overall density of CD8+ TILs varied from 1-50% (median: 5%; high overall CD8+ TILs: 10%, 48/125), whereas overall PD1+ TILs ranged from 0-60% (median: 3%; high overall PD1+ TILs: 4%, 49/125) (Supplementary Figure 1).

### Expression of PD-L1

Immunohistochemically, at least partial membranous PD-L1 staining was detectable in 71% of ESCCs (89/125, median: 3%), showing a range from 0-90% positive tumor cells. 38/125 (30%) cases were classified as PD-L1high as their amount of PD-L1 positive TCs exceeded the 66. percentile (>10% positive TCs). Weak (34%), intermediate (31%) and strong (35%) staining intensities were almost evenly distributed among the PD-L1 positive cases.

PD-L1+ TILs were detectable in 109/125 cases (87%) and their density within the whole tumor area varied from 0-60% (median: 3%; high overall PD-L1+ TILs: 5%, 36/125) (Table 1, Figure 1).

### *PD-L1* copy number status

Assessment of the *PD-L1* copy number status via FISH revealed *PD-L1* amplifications in 3/125 (2%) and *PD-L1* deletions in 10/125 (8%) ESCCs. The remaining cases displayed *PD-L1* disomy (92/125; 74%) or polysomy (19/125, 15%) (Table 1, Supplementary Figure 1).

### Subgrouping of ESCCs

Slightly modifying the recently proposed immune-based cancer classification system by Teng et al. [13], ESCCs were stratified into 4 different subgroups based on their CD3i status and their amount of PD-L1 positive TCs. The resulting four subgroups were the following: subgroup 1: CD3ihigh / PD-L1high (27/125; 21.6%); subgroup 2: CD3ilow / PD-L1low (60/125; 48.0%); subgroup 3: CD3ilow / PD-L1high (21/125; 16.8%); subgroup 4: CD3ihigh / PD-L1low (17/125; 13.6%).

### Correlation of immunological factors with each other

As highlighted in Table 2, CD3ihigh and CD8ihigh cases were each significantly associated with diffuse CD3i/CD8i distribution (p<0.001, respectively) and PD1ihigh - (p<0.001, respectively) as well as PD-L1high tumors (CD3: p<0.001; CD8: p=0.005). Additionally, diffusely distributed CD3i and CD8i tumors were significantly more frequent in cases with PD1ihigh - (CD3: p<0.001; CD8: p=0.014) and PD-L1high tumors (CD3: p<0.001; CD8: p=0.003). A high PD1i count was not associated with PD-L1high tumors. CD3i/CD8i/PD1i count correlated with the overall density of TILs of their respective subpopulation (CD3: p=0.002; CD8: p<0.001; PD1: p<0.001). Details of the correlations of overall TIL density are given in Supplementary Table 1.

**Table 2:** Rank-order correlations of intraepithelial lymphocytes with immunological factors in ESCC

	High CD3i	High CD8i	Diffuse CD3i	Diffuse CD8i	High PD1i	High PD-L1+ TILs	PD-L1 high
	>20 CD3+ TILs/100 TCs	>13 CD8+ TILs/100 TCs			>6 PD-1+ TILs/100 TCs		>10% PD-L1+ TCs
**High CD3i**							
>20 CD3+ TILs/100 TCs	x	*r* = 0.642	*r* = 0.595	*r* = 0.392	*r* = 0.456	*r* = 0.091	*r* = 0.348
		*p* < 0.001	*p* < 0.001	*p* < 0.001	*p* < 0.001	*p* = 0.409	*p* < 0.001
**High CD8i**							
>13 CD8+ TILs/100 TCs		x	*r* = 0.419	*r* = 0.591	*r* = 0.442	*r* = 0.093	*r* = 0.265
			*p* < 0.001	*p* < 0.001	*p* < 0.001	*p* = 0.533	*p* = 0.005
**Diffuse CD3i**							
			x	*r* = 0.385	*r* = 0.326	*r* = 0.09	*r* = 0.363
				*p* < 0.001	*p* < 0.001	*p* = 0.072	*p* < 0.001
**Diffuse CD8i**							
				x	*r* = 0.265	*r* = 0.056	*r* = 0.269
					*p* = 0.014	*p* = 0.533	*p* = 0.003
**High PD1i**							
>6 PD-1+ TILs/100 TCs					x	*r* = 0.193	*r* = 0.170
						*p* = 0.04	*p* = 0.081
**High PD-L1+ TILs**							
						x	*r* = 0.152
							*p* = 0.1

#### Correlation of immunological factors with clinicopathologic variables

CD3ihigh tumors were significantly associated with lower pT stage (p=0.01), lower UICC stage (p=0.001) and tumor free local lymph nodes (p=0.001). Cases showing a diffuse distribution of CD3is were also more frequent in lower pT stage (p=0.02) and lacked nodal involvement more often (p=0.03). PD1ihigh tumors showed lower pT stage (p=0.015) and frequently lacked nodal involvement (p=0.02). CD8ihigh and PD-L1high status, PD-L1 expression in TILs and *PD-L1* copy number status were not associated with clinicopathologic variables. Overall density of TILs across the tumor area (including TCs and tumor stroma) showed no significant associations with clinicopathologic features.

#### Correlation of clinicopathological factors with survival parameters

UICC stage (OS: p=0.008; DSS: p=0.002; DFS: p<0.001), pT (OS: : p<0.001; DSS: : p<0.001; DFS: p=0.002), pN (OS: p=0.012; DSS: p=0.014; DFS: p=0.005) and pM (OS: p=0.001; DSS: p<0.001; DFS: p<0.001) stadium were all significantly associated with survival parameters, while conventional histopathologic grade only displayed a comparatively weak association with OS (p=0.039), but not with DSS or DFS. Age and sex played no role in survival prediction (Table 1).

#### Correlation of immunological factors with survival parameters and clinical outcome

**Figure 2:**
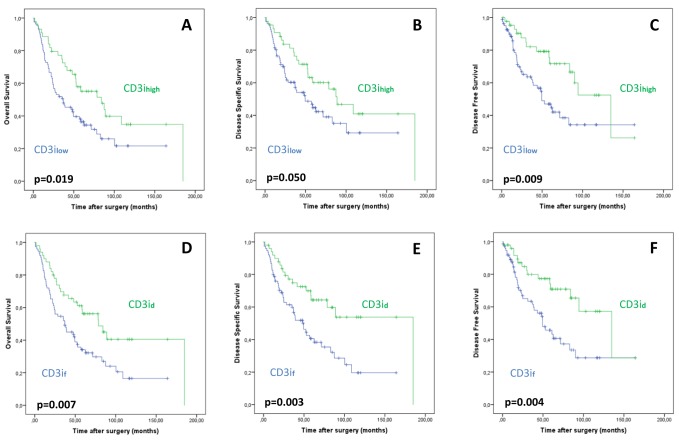
Association of CD3ihigh status with improved overall **A**., disease-specific **B**. and disease-free **C**. survival. Correlation of diffuse (CD3id) and focal (CD3if) distribution of CD3i on overall **D**., disease-specific **E**. and disease-free **F**. survival.

**Figure 3:**
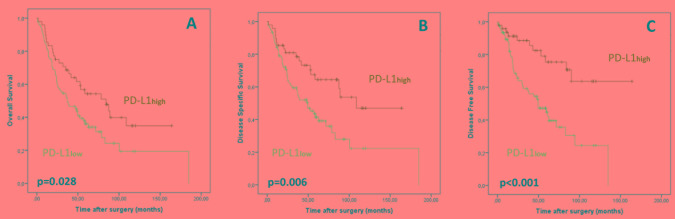
Association of PD-L1high status with improved overall **A**., disease-specific **B**. and disease-free **C**. survival.

As demonstrated in Table 1 and Figure 2, univariate analysis revealed that patients with CD3ihigh tumors showed favorable OS (p=0.019), DSS (p=0.05) and DFS (p=0.009) compared to patients with low CD3is. While patients with CD3ihigh tumors displayed a mean OS of 96.4 months (DSS: 106.1 months; DFS: 104.9 months), mean OS (62.1 months) in cases harboring low CD3is was significantly shorter (DSS: 73.9 months; DFS: 78.1 months). Additionally, ESCC-patients with diffusely distributed CD3is displayed favorable OS (p=0.007), DSS (p=0.003) and DFS (p=0.004) (Figure 2). PD1ihigh cancers were associated with favorable OS (p=0.035). Pure CD8i count did not show prognostic impact. Overall density of TILs across the tumor area (including tumor cells and tumor stroma) had no impact on prognosis. As highlighted in Figure 3, PD-L1high ESCCs displayed improved OS (p=0.028), DSS (p=0.006) and DFS (p<0.001), while the extent of PD-L1+ TILs, PD-L1 staining intensity in TCs and *PD-L1* copy number status were not associated with survival parameters. Multivariate survival analyses (including gender, age, pT, pN) revealed improved survival parameters for CD3ihigh tumors (OS: p=0.011; DSS: p=0.045; DFS: p=0.004), diffusely distributed CD3is (OS: p=0.01; DSS: p=0.005; DFS: p=0.009), diffusely distributed CD8is (OS: p=0.005; DSS: p=0.03; DFS: p=0.01) and PD-L1high ESCCs (OS: p=0.026; DSS: p=0.009; DFS: p=0.001). Details on multivariate survival analyses are given in Table 3 and Supplementary Table 2.

**Table 3:** Multivariate analysis of the impact of CD3i – and PD-L1 status on overall survival

		HR (OS)	lower CI (95%)	upper CI (95%)	*p*-value
Gender	*male*	1.000			*0.026*
	*female*	0.518	0.290	0.925	
					
Age	*per year*	1.022	0.994	1.051	*0.121*
pT	*1*	1.000			*<0.001*
	*2*	3.345	1.918	5.834	
	*3*	1.693	0.875	3.276	
	*4*	2.068	0.223	19.211	
pN	*0*	1.000			*0.038*
	*1*	1.441	0.879	2.361	
	*2*	0.554	0.149	2.057	
	*3*	6.048	1.288	28.387	
CD3i	*high*	1.000			*0.011*
	*low*	1.925	1.159	3.200	
		HR (OS)	lower CI (95%)	upper CI (95%)	p-value
Gender	*male*	1.000			*0.044*
	*female*	0.551	0.308	0.985	
					
Age	*per year*	1.026	0.997	1.056	*0.077*
pT	*1*	1.000			*0.001*
	*2*	3.025	1.736	5.272	
	*3*	1.723	0.893	3.325	
	*4*	2.962	0.343	25.556	
pN	*0*	1.000			*0.040*
	*1*	1.471	0.892	2.426	
	*2*	0.602	0.168	2.163	
	*3*		5.952	1.269	*27.904*
CD3i	*diffuse*	1.000			*0.010*
Distribution	*focal*	1.941	1.173	3.210	
		HR (OS)	lower CI (95%)	upper CI (95%)	p-value
Gender	*male*	1.000			*0.088*
	*female*	0.597	0.329	1.080	
Age	*per year*	1.022	0.995	1.049	*0.112*
pT	*1*	1.000			*<0.001*
	*2*	3.415	1.978	5.895	
	*3*	1.956	1.013	3.777	
	*4*	5.081	0.529	48.760	
pN	*0*	1.000			*0.048*
	*1*	1.248	0.773	2.014	
	*2*	0.388	0.105	1.432	
	*3*	5.210	1.110	24.457	
PD-L1 TCs	*high*	1.000			*0.026*
	*low*	1.801	1.074	3.021	

**Figure 4:**
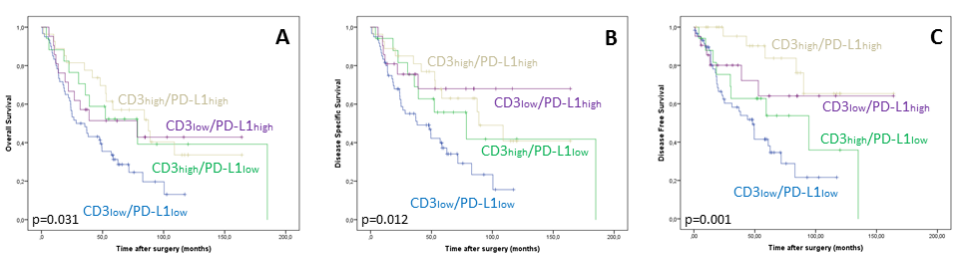
Decreased overall **A**., disease specific **B**. and disease free survival **C**. of the CD3ilow / PD-L1low ESCC subgroup.

Additional ESCC subgrouping based on T-cell infiltration (CD3i status) and PD-L1 status revealed the CD3ilow / PD-L1low subgroup to be significantly associated with reduced OS (p=0.031), DSS (p=0.012) and DFS (p=0.001) compared to the other subgroups. Mean OS (DSS; DFS) of patients with CD3ilow/PD-L1low ESCC was 46.8 months (51.6 months; 53.8 months) compared to 76.9 months (117.4 months; 113.9 months) for patients with CD3ilow / PD-L1high tumors, 86.4 months (100.6 months; 130.3 months) for patients with CD3ihigh / PD-L1high cancers and 95.4 months (101.4 months; 77.4 months) for patients with CD3ihigh / PD-L1low ESCCs (Table 1; Figure 4). The observed impact on survival was confirmed by a subsequent multivariate analysis including age, gender and stage (OS p=0.031; DSS p=0.017; DFS p=0.002; Table 3; Supplementary Table 2).

## DISCUSSION

In this study, we analyzed the composition of the IM in a comparatively large cohort of 125 primary resected, therapy-naive ESCCs and demonstrate CD3ihigh - as well as PD-L1high tumors not only to be significantly associated with one another, but also to be independent prognosticators of a beneficial ESCC disease course in uni- and multivariate statistical analyses.

Furthermore, an additional, immune-based ESCC stratification demarcated a prognostically unfavorable subgroup of CD3ilow / PD-L1low cancers.

High densities of CD3+ / CD8+ TILs have been associated with improved survival and a favorable disease course in various cancers [8–11, 14, 15, 27, 28] and high CD3i levels have been identified as strong and independent prognosticators for example in human colorectal [14] and ovarian cancer [16, 17]. CD3, a transmembranous protein virtually exclusive to the T-cell lineage, stains all T-cell subgroups, represents the gold standard for the assessment of overall T-cell infiltration in daily clinicopathological practice [29] and can therefore, in our opinion, be used to determine whether a given cancer is in the state of a so called “T - cell inflamed microenvironment” or not, a condition which has been postulated to be of crucial importance for the efficacy of immune checkpoint inhibitors [12, 13, 30]. In line with these findings, our data show a high count and a diffuse distribution of CD3is, but not of overall CD3+ TILs, to be prognostic of favorable OS, DSS and DFS in ESCC, suggesting that the assessment of CD3is is a comparatively specific and standardized approach to evaluate the T-cell mediated tumor host relationship, hypothesizing that intraepithelial lymphocytes are more likely to truly interact with cancer cells than their stromal counterparts. Surprisingly, to our knowledge, the prognostic and clinicopathologic influence of overall CD3+ TILs and especially of CD3is has not yet been studied in ESCC, as the few, and rather loosely connected studies regarding TILs in ESCC rather focused their efforts on studying potential effects of the PD-1 / PD-L1 axis [21, 24] or overall CD8+ TILs [21]. Interestingly, in contrast to previous work in ESCC [21], neither pure CD8i- or overall CD8+ TIL count displayed predictive relevance in our cohort, emphasizing that the complex interplay between distinctive T-cell subgroups and cancer cells is not sufficiently recognized through detection of CD8+ TILs alone [31].

Data on expression rates and prognostic value of PD-L1 immunohistochemistry in ESCC is rather scarce and partially conflicting, as some studies associate PD-L1 positive ESCC with poor patient outcome [24, 25, 32], while two recent studies [21, 22] demonstrated PD-L1 positivity to be predictive of a rather favorable disease course. Pointing in the same direction, our data, generated with a robust and highly specific PD-L1 (SP263) antibody [33] [34–36] in a comparatively large and extensively investigated, therapy naive tumor series, associate PD-L1high ESCC with favorable OS, DSS and DFS survival in uni- and multivariate analyses. However, comparability between ESCC studies is either hampered by small cohort sizes [25], varying PD-L1 antibodies and cutoffs, or differences in the amount of investigated cancerous tissue [21, 24, 25, 32]. Noteworthy, *PD-L1* copy number analysis revealed *PD-L1* amplifications to be a far less frequent event in ESCC (2%) than in squamous cell carcinoma of the oral cavity (19%) [37], indicating an intertumoral spread width regarding the biologic mechanisms underlying PD-L1 expression between primary squamous cell carcinomas of different localizations [38]. Furthermore, PD-L1 staining intensity showed no predictive value in our ESCC tumor series.

Besides in ESCC, the association of immunohistochemical PD-L1 expression to a beneficial outcome has been observed in several entities like colorectal carcinoma [39], non-small cell lung cancer [40], melanoma [41], Merkel cell carcinoma [42] or breast cancer [43], although the mechanisms underlying the observed positive effects remain poorly understood. Nevertheless, as PD-L1high ESCC was significantly associated with a concurrent CD3ihigh, CD8ihigh and PD1ihigh status in our cohort, one might hypothesize that high PD-L1 expression in ESCC might rather be interpreted as an adaptive mechanism of a given cancer in response to an immunoactive tumor-host relationship [41], that could therefore contribute to an improved disease course and speculatively, to a potential response towards immune checkpoint therapy [44].

Taking our analyses one step further, we stratified our ESCC cohort into four immunogenic subgroups based on their PD-L1 / CD3i status. Interestingly, our subgrouping approach, which represents a slightly adjusted version of the TIL/PD-L1 based cancer classification proposed by Teng et al. [13], unmasked a comparatively large subgroup of “non-immunogenic”, CD3ilow / PD-L1low ESCCs (48% of all tumors), which were significantly associated with reduced survival parameters in uni- and multivariate analyses, while certain tendencies, but no distinct differences in patient survival were observed between the other subgroups. Although no clear prognostic separation of the CD3ihigh / PD-L1high subgroup was visible, we believe that these stratification data are concordant with our general findings and with those from certain previous studies [21, 22], as they prognostically segregate those tumors that show at least partial immunoreactivity in a certain manner from those who find themselves in a completely “non-immunogenic” state.

Our work has some limitations: (1) our investigations were performed on TMA-basis, although we examined a substantial number of tumor cores deriving from the diverse tumor compartments (apical and central tumor region, invasive margin). Furthermore (2), only resection specimens (3) without postoperative checkpoint blockade were investigated. As our work is retrospective in nature, our data need to be validated in larger, prospective ESCC cohorts as well as on biopsy material. Furthermore, the suitability of PD-L1 IHC, intraepithelial CD3+ TILs and the subsequent immunologic ESCC subgroups as a potential rationale for immune checkpoint therapy regimens clearly needs to be investigated by subsequent clinical studies in appropriate patient cohorts.

Summarizing, we investigated the contexture of ESCCs IM and demonstrate increased intraepithelial CD3+ TILs and high PD-L1 expression on tumor cells to be independent predictors of a beneficial clinical outcome in ESCC. This study not only highlights the comparatively frequent expression of PD-L1 in this tumor entity and underlines the close association of PD-L1 positive ESCCs with increased numbers of intraepithelial T-lymphocytes, but also identifies a subset of non-immunogenic ESCCs with a distinct clinical course, potentially forming the basis for an immune-based stratification in this tumor entity.

## MATERIALS AND METHODS

### Cohort recruitment

Our tumor series comprised 125 ESCC patients, who underwent surgical resection between 1994 and 2007 at Klinikum Rechts der Isar of the Technical University of Munich, Germany and at the University Hospital Heidelberg, Germany. All tumors were chemo-/radiation-naive at the time of resection and none of the patients received immune checkpoint inhibitors or underwent other immune therapy regimens. Only tissue from primary tumors was investigated. Hematoxylin-Eosin (H&E) stained sections were initially reviewed by two pathologists (MJ, MB), who confirmed the diagnosis of ESCC. Grading and staging of ESCC at the time of diagnosis was performed according to the current World Health Organization Classification of Tumors of the Digestive System [26] and the UICC tumor, node, metastasis (TNM) classification [45]. Additional clinicopathologic characteristics (sex, age, tumor localization, local, nodal / distant relapse) and established survival parameters such as overall survival (OS), disease-specific survival (DSS) and disease-free survival (DFS) were collected for all patients. This study has been approved by the Ethics Review Committee of the Technical University of Munich (503/16 S).

### Tissue microarray construction

Formalin-fixed paraffin-embedded (FFPE) tumor samples from the central and apical tumor region as well as from the invasive margin were assembled into a tissue microarray (TMA) using a Tissue Microarrayer (Beecher Instruments, Sun Praierie, USA) with a core size of 0.6 mm. All samples of a respective tumor region (central/apical tumor region, invasive margin) were extracted from areas harboring a high tumor/stroma ratio. Mean tumor cell content per core was 79.3%, ranging from 40% - 99 %. Obvious inflammatory hotspots (such as lymph follicles or areas of ulceration) were avoided. Considering tumor heterogeneity, a minimum of 3 and (where feasible) up to 6 tumor cores were taken from the primary tumors in areas previously marked by five pathologists (MJ, MB, JS, ED, RL).

### Immunohistochemistry

IHC was performed on 2 μm sections from each TMA using a PD-L1 primary antibody (VENTANA, clone (c): SP-263; dilution (d): 1:100), a PD-1 primary antibody (Cell Marque, c: 11RQ-22, d: 1:50) and primary antibodies against CD3 (Cell Marque, c: MRQ39, d: 1:500) and CD8 (DAKO, c: C8/144B, d: 1:50), using an automated immunostainer with an iVIEW DAB detection kit (Ventana Medical Systems, Roche, Mannheim, Germany).

### Scoring of PD-1+, CD3+ and CD8+ tumor infiltrating lymphocytes

Scoring of TILs (PD-1+, CD3+, CD8+) was performed jointly by two pathologists (MJ, MB) blinded to clinicopathological outcome. In general, TILs were separately evaluated in every core of the TMA and an average score resulting from all cores (and all tumor regions) was assigned as the final TIL count for the respective case. The analysis of TIL- subpopulations was performed in two ways: (1) Scoring of TILs was performed in the tumor region of the respective core showing the highest density of the particular TIL subpopulation on low power magnification (4x). Within this region, the amount of intraepithelial CD3+ TILs (CD3i), intraepithelial CD8+ TILs (CD8i) and intraepithelial PD-1+ TILs (PD1i) was scored manually by counting the quantity of the respective lymphocyte subpopulation within tumor cell clusters of 100 tumor cells using high power magnification (40x). (2) In analogy to previous TIL-scoring approaches [46, 47] overall density of TILs was evaluated via determination of the percent proportion of the tumor area occupied by the respective TIL subpopulations. The tumor area was defined as tumor cells (TCs) and tumor stroma, while areas of tumor necrosis or inflamed peritumoral areas were not taken into account. All corresponding tumor cores of each case were analyzed and the average density across all cores was calculated. The infiltration pattern of (CD3+/CD8+) TILs was classified as diffuse, if the respective neoplasms showed a continuous intraepithelial infiltration, whereas a patchy, discontinuous infiltrate was reported as focal.

### Immunohistochemical scoring of PD-L1 in tumor cells and tumor infiltrating lymphocytes

PD-L1 expression in TCs and in TILs was jointly determined by two pathologists (MJ, MB) and was evaluated separately in every TMA core before a final score resulting from all investigated cores (and all tumor regions) was assigned. In TCs, the absolute percentage of positive cells was determined. Only membranous staining patterns were scored as positive. The intensity of PD-L1 staining was scored using a 4-tiered grading system established in a recent study regarding the efficacy of the used PD-L1 SP263 antibody [33], including “no staining” (0), “weak staining” (1+), “intermediate staining” (2+) and “strong staining” (3+). Immunohistochemical expression of PD-L1 in TILs was assessed via a determination of the percent proportion of the tumor area occupied by PD-L1+ TILs, as described previously [47]. Since no obvious differences in staining intensity were noted, staining intensity of PD-L1 in TILs was not taken into account.

### Determination of immunohistochemical scoring groups / cut offs

Following the general scoring process, the respective data were quantified regarding their respective percentile value and patients were stratified into three subgroups: lower (below 33. percentile), intermediate (33.-66. percentile) and upper third (exceeding 66. percentile). For further analyses, cases within the upper third were classified as “high”, whereas all cases ranging within the intermediate or the lower third were classified as “low”.

### *PD-L1* Fluorescence in situ Hybridization

FFPE tissue sections (thickness 2μm) of TMAs were used for dual-color FISH analysis using a SPEC *CD274, PDCD1LG2/CEP*9 dual color probe and *PD-L1* copy number status (CNS) was evaluated as described previously counting at least 20 tumor cell nuclei per tumor core [37]. *PD-L1* amplification was defined as *PD-L1/CEP9* ratio ≥2.0, with high level amplification defined as ≥4.0 and low level amplification defined as ≥2.0 and ≤4.0. Polysomy 9 was defined as average *PD-L1* copy number >3 signals/cell. *PD-L1* deletion was defined as *PD-L1/CEP9* ratio <0.8.

### Statistical analyses

Statistical analyses were performed using SPSS 23 (SPSS Inc, Chicago, IL, USA). Correlations between immunologic characteristics and clinicopathological parameters were calculated using Χ^2^ test and Fisher's exact test. Associations of immunologic factors with each other were calculated with Spearman's rank order correlation. Survival probabilities were plotted using the Kaplan-Meier method and a log-rank test was used to probe the significance of differences in survival probabilities. Multivariate survival analysis was performed utilizing the Cox proportional hazard model. All significances were two-sided, p-values ≤0.05 were considered significant.

## SUPPLEMENTARY MATERIALS FIGURE AND TABLE



## Abbreviations

CD3ihigh: High intraepithelial CD3+ tumor infiltrating lymphocytes
CD8ihigh: High intraepithelial CD8+ tumor infiltrating lymphocytes
DSS: Disease specific survival
DFS: Disease free survival
ESCC: Esophageal squamous cell carcinoma
FISH: Fluorescence in situ Hybridization
FFPE: Formalin-fixed paraffin-embedded
IM: immunogenic microenvironment
OS: Overall survival
PD1ihigh: High intraepithelial PD1+ tumor infiltrating lymphocytes
PD1: Programmed Death-1
PD-L1: Programmed Death – Ligand 1
TC: Tumor cells
TIL: Tumor infiltrating lymphocyte
TMA: Tissue micro array
