# Supplementary material for: Increased intraepithelial CD3+ T-lymphocytes and high PD-L1 expression on tumor cells are associated with a favorable prognosis in esophageal squamous cell carcinoma and allow prognostic immunogenic subgrouping
Source: Oncotarget. 2017 Jun 22;8(29):46756–68. doi: 10.18632/oncotarget.18606 (PMC5564521; doi:10.18632/oncotarget.18606)
Supplement: Supplementary file 1 [file oncotarget-08-46756-s001.pdf]

Increased intraepithelial CD3+ T-lymphocytes and high PD-L1 expression on tumor cells are associated with a favorable prognosis in esophageal squamous cell carcinoma and allow prognostic immunogenic subgrouping

Supplementary Material

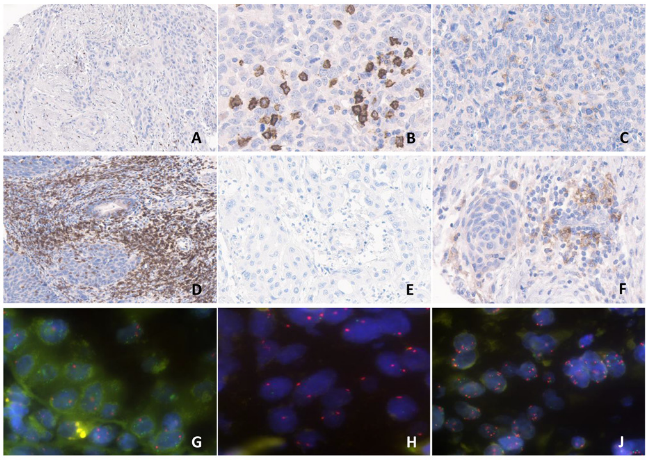

Supplementary Figure 1: Low (**A**) and high rates (**B**) of overall TILs (CD3+); low (**C**) and high (**D**) CD8i count; (**E**) high PD1i count; (**F**) overall PD-L1+ TILs; PD-L1 copy number status showing an (**G**) amplification, (**H**) a deletion and a polysomy (**J**).

Supplementary Table 1: Rank-order correlations of overall TIL densities.

	High CD3	High CD3i	High CD8	High CD8i	High PD-1	High PD-1i	High PD-L1+ TILs	*PD-L1* CNV	PD-L1 high (tumor)
**High CD3**	x	r=0.279 p=0.002	r=0.357 p<0.001	r=0.193 p=0.031	r=0.252 p=0.006	0.304 p=0.001	r=0.370 p<0.001	r=0.044 p=0.567	r=0.324 p<0.001
**High CD8**		r=0.417 p<0.001	x	r=0.551 p<0.001	r=0.321 p<0.001	r=0.442 p<0.001	r=0.261 p=0.004	r=-0.082 p=0.046	r=0.256 p=0.004
**High PD-1**		r=0.263 p=0.004		r=0.326 p<0.001	x	r=0.793 p<0.001	r=0.307 p=0.001	r=0.108 p=0.597	r=0.118 p=0.197

Supplementary Table 2: Multivariate analysis of the impact of CD3i / CD8i distribution and CD3i / PD-L1 subgroups on disease specific- and disease free survival.

		HR (DSS)	lower CI	Upper CI	p-value	HR (DFS)	lower CI	Upper CI	p-value
Age	*per year*	1,032	1,002	1,062	*0,037*	1,018	0,984	1,053	*0,309*
Gender	*male*	1,000			*0,048*	1,000			*0,329*
	female	0,496	0,247	0,995		0,688	0,324	1,46	
pN	*0*	1,000			*0,059*	1,000			*0,103*
	1	1,143	0,667	1,959		1,261	0,694	2,292	
	2	0,419	0,11	1,592		0,731	0,237	2,256	
	3	6,059	1,245	29,479		6,287	1,255	31,503	
pT	*1*	1,000			*<0,001*	1,000			*0,001*
	2	4,009	2,156	7,458		4,157	2,09	8,266	
	3	2,228	1,054	4,708		2,194	0,978	4,924	
	4	7,244	0,709	74,005		censored			
PD-L1 TCs	*high*	1,000			*0,009*	1,000			*0.001*
	low	2,238	1,225	4,09		3,44	1,667	7,099	

		HR (DSS)	lower CI	Upper CI	p-value	HR (DFS)	lower CI	Upper CI	p-value
Age	*per year*	1,033	1,002	1,065	*0,035*	1,022	0,986	1,058	*0,234*
Gender	*male*	1,000			*0,009*	1,000			*0,063*
	female	0,405	0,205	0,801		0,496	0,237	1,037	
pN	*0*	1,000			*0,048*	1,000			*0,05*
	1	1,317	0,755	2,295		1,522	0,812	2,854	
	2	0,647	0,169	2,477		1,472	0,485	4,47	
	3	7,665	1,576	37,285		9,035	1,774	46,021	
pT	*1*	1,000			*<0,001*	1,000			*0,002*
	2	3,938	2,098	7,392		3,927	1,942	7,942	
	3	1,914	0,908	4,032		1,645	0,722	3,746	
	4	2,397	0,246	23,306		censored			
CD3I	*high*	1,000			*0,045*	1,000			*0,004*
	low	1,778	1,013	3,122		2,571	1,341	4,928	

		HR (DSS)	lower CI	Upper CI	p-value	HR (DFS)	lower CI	Upper CI	p-value
Age	*per year*	1,038	1,006	1,072	*0,021*	1,025	0,988	1,064	*0,189*
Gender	*male*	1,000			*0,017*	1,000			*0,107*
	female	0,435	0,219	0,862		0,545	0,261	1,14	
pN	*0*	1,000			*0,054*	1,000			*0,049*
	1	1,379	0,784	2,424		1,569	0,832	2,961	
	2	0,748	0,203	2,761		1,505	0,495	4,58	
	3	7,458	1,531	36,334		8,878	1,747	45,106	
pT	*1*	1,000			*0,002*	1,000			*0,006*
	2	1,893	0,898	3,988		3,431	1,714	6,871	
	3	3,517	1,868	6,622		1,689	0,746	3,826	
	4	3,492	0,392	31,14		censored			
CD3I	*diffuse*	1,000			*0,005*	1,000			*0,009*
Distribution	*focal*	2,267	1,273	4,035		2,348	1,237	4,458	

		HR (DSS)	lower CI	Upper CI	p-value	HR (DFS)	lower CI	Upper CI	p-value
Age	*per year*	1,038	1,007	1,07	*0,017*	1,027	0,992	1,063	*0,136*
Gender	*male*	1,000			*0,003*	1,000			*0,018*
	female	0,345	0,172	0,694		0,399	0,186	0,853	
pN	*0*	1,000			*0,076*	1,000			*0,090*
	1	1,221	0,71	2,099		1,325	0,725	2,422	
	2	0,633	0,165	2,423		1,358	0,452	4,08	
	3	6,875	1,415	33,409		7,712	1,536	38,725	
pT	*1*	1,000			*<0,001*	1,000			*0,001*
	2	3,902	2,097	7,264		4,09	2,051	8,154	
	3	2,061	0,981	4,332		1,959	0,875	4,383	
	4	2,370	0,243	23,083		censored			
CD8I	*diffuse*	1,000			*0,030*	1,000			*0,010*
Distribution	*focal*	1,882	1,063	3,332		2,397	1,236	4,650	
